# Human Liver MSCs Retain Their Basic Cellular Properties in Chronically Inflamed Liver Tissue

**DOI:** 10.3390/ijms252413374

**Published:** 2024-12-13

**Authors:** Yan S. Kim, Alexey Yu. Lupatov, Veronika V. Burunova, Nikolay N. Bagmet, Nikita K. Chardarov, Svyatoslav L. Malov, Roman V. Kholodenko, Garnik A. Shatverian, Garik V. Manukyan, Konstantin N. Yarygin, Irina V. Kholodenko

**Affiliations:** 1 Laboratory of Cell Biology, V.N. Orekhovich Institute of Biomedical Chemistry, 119121 Moscow, Russiakyarygin@yandex.ru (K.N.Y.); 2 Department of Abdominal Surgery and Oncology, Laboratory of Emergency Surgery and Portal Hypertension, Petrovsky National Research Centre of Surgery, 119435 Moscow, Russia; 3 Laboratory of Molecular Immunology, Shemyakin-Ovchinnikov Institute of Bioorganic Chemistry, Russian Academy of Sciences, 117997 Moscow, Russia; 4 Department of General Pathology and Pathophysiology, Russian Medical Academy of Continuous Professional Education, 125284 Moscow, Russia

**Keywords:** liver mesenchymal stem cell, cirrhosis, fibrosis, cell phenotype, proliferation, migration, cellular senescence, secretome, cell therapy

## Abstract

Every 25th death worldwide is associated with liver pathology. The development of novel approaches to liver diseases therapy and protocols for maintaining the vital functions of patients on the liver transplant waiting list are urgently needed. Resident mesenchymal stem cells (MSCs) play a significant role in supporting liver tissue integrity and improve the liver condition after infusion. However, it remains unclear whether MSCs isolated from chronically inflamed livers are similar in their basic cellular properties to MSCs obtained from healthy livers. We applied a large array of tests to compare resident MSCs isolated from apparently normal liver tissue and from chronically inflamed livers of patients with fibrosis, cirrhosis, and viral hepatitis. Chronic inflammatory environment did not alter the major cellular characteristics of MSCs, including the expression of MSC markers, stem cell markers, adhesion molecules, and the hallmarks of senescence, as well as cell proliferation, migration, and secretome. Only the expression of some immune checkpoints and toll-like receptors was different. Evidently, MSCs with unchanged cellular properties are present in human liver even at late stages of inflammatory diseases. These cells can be isolated and used as starting material in the development of cell therapies of liver diseases.

## 1. Introduction

The liver is a vital organ that has unique detoxifying and regenerative properties. However, viruses, alcohol, unhealthy food, toxicants, and other factors continually damage hepatocytes and the endothelial barrier, leading to the abundant tissue infiltration by the inflammatory cells, as well as massive accumulation of the extracellular matrix due to excessive collagen production by myofibroblasts derived from activated hepatic stellate cells. These pathological processes initiate the development of liver fibrosis, which in the later stages progresses to cirrhosis and the end-stage liver disease. Liver failure occurs when damage exceeds the compensatory capacity of the liver. Organ transplantation is the only available and effective treatment for the end-stage liver disease. However, not all patients can receive such treatment in a timely manner due to the limited organ transplant supply [1]. Patients on the waiting list receive palliative care, the main aim of which is to relieve symptoms, pain and stress [2]. Unfortunately, such assistance is often ineffective, and many patients fail to survive until actual transplantation. Therefore, along with palliative care, additional methods are needed to effectively maintain the liver in a functional state in order to guide patients throughout the waiting period. Cell therapy is one of the novel approaches under development. In particular, certain successes have been achieved in the treatment of fibrosis and cirrhosis using mesenchymal stem cells (MSCs) transplantation. The safety of the treatment of liver pathologies with MSCs transplantation has been demonstrated in many clinical trials, most of which also showed an improvement in the synthetic function of the liver, as well as its partial histological restoration [3]. For example, transplantation of allogeneic bone marrow-derived MSCs to patients with hepatitis B virus (HBV)-related acute-on-chronic liver failure increased the cumulated survival rate and caused a significant improvement in clinical parameters (including total serum bilirubin and Model for End-Stage Liver Disease scores), and decreased the incidence of severe infection in the MSC group compared to the control group. Concerning the side effects, a higher incidence of fever in the group of patients receiving allogeneic MSC infusions compared to the control group was noted [4]. Patients with HBV-related decompensated liver cirrhosis who received allogeneic umbilical cord MSC infusion also showed a significantly higher overall survival rate and improved liver function as indicated by serum albumin, prothrombin activity, cholinesterase, and total bilirubin levels during 48 weeks of observation. No side effects associated with allogeneic MSC infusion were identified [5,6,7]. Results of phase II clinical trials aimed at assessing the efficacy of allogeneic liver-derived progenitor cells (HepaStem) infusion in patients with acute liver failure associated with chronic and acute decompensation have been published. Overall, markers of systemic inflammation and altered liver function gradually decreased in surviving patients treated with the liver-derived MSCs. Survival rates at day 28 and month 3 were 83% (20/24) and 71% (17/24), respectively, and no patient had acute-on-chronic liver failure at month 3 [8]. Furthermore, infusion of allogeneic or autologous MSCs to patients undergoing liver transplantation resulted in a decrease in the incidence of the antibody-mediated rejection and acute cellular rejection [9], caused moderate positive changes in the peripheral blood immunoregulatory T and NK cells [10], and had a pronounced overall immunosuppressive effect [11], indirectly suggesting the development of the immunological tolerance to the transplant.

MSCs can be isolated from various tissue sources, including the liver [12]. It is essential to select the source of MSCs which are the most effective in maintaining the pathological liver in a functional state. It has been suggested that resident tissue MSCs are committed to their source of origin. Liver MSCs are no exception in this context and, along with the standard characteristics of MSCs, also exhibit certain properties characteristic of liver tissue; in particular, they express a number of hepatocyte-specific genes and differentiate into hepatocytes more effectively compared to umbilical cord MSCs [13]. It is likely that liver-derived MSCs may be more effective in the treatment of liver pathologies than MSCs from other tissue sources. However, in this case, the use of allogeneic cells seems most appropriate. Autologous transplantation might appear challenging because of the severe inflammatory condition of the organ, which may affect resident MSCs. However, it is not the case, and if a biopsy could be taken from such patients, the isolation of MSCs and their expansion in vitro was not difficult [14,15,16].

Since chronic inflammation plays an important role in liver pathophysiology and non-parenchymal stromal/mesenchymal cells, which normally play a supporting role in liver homeostasis, are activated under inflammatory conditions [17], MSC-based therapy isolated from healthy non-inflamed tissue sources has been proposed as a promising strategy for the restorative treatment of liver diseases. In his review, Krampera [18] clarified that in the absence of “licensing”, MSCs are not constitutively immunosuppressive, and their immunomodulatory properties depend on the local inflammatory environment they enter after transplantation. Indeed, inflammation is an important regulator of stem cells, which can respond to inflammatory cascades and participate in them directly. For example, stem cells express receptors that recognize PAMPs (pathogen-associated molecular patterns) and DAMPs (damage-associated molecular patterns), which are the initial triggers of the inflammatory response. In response to inflammation, stem cells are able to mobilize, proliferate, and secrete cytokines that further enhance the response [19]. MSCs, like other stem cells, respond to environmental signals by adapting their fate and functions, leading to their migration, proliferation, and/or tissue regeneration [20]. Therefore, it is important to understand which sources of MSCs are potentially suitable for use in cell therapy and how they may respond to the inflammatory microenvironment.

The aim of this work was to evaluate the basic cellular characteristics of MSCs isolated from normal and pathological liver, including phenotype, proliferative potential and migration abilities in vitro, and cellular senescence. The need to assess such basic characteristics is important for further understanding the potential of liver MSCs in cell therapy of liver diseases, since at the first stage, the ability to obtain cells from tissue, their expansion in vitro, high survival during cultivation, preservation of migration properties, and weak senescence are important. Moreover, the question whether MSCs isolated from healthy liver differ from MSCs isolated from pathological liver remains open. To resolve this problem, we derived and compared MSCs isolated from normal liver (Normal Liver MSCs, NL-MSCs) and MSCs isolated from pathological liver (Pathological Liver MSCs, PL-MSCs) affected by severe diseases with system inflammation processes, such as cirrhosis, fibrosis, or virus hepatitis. NL-MSCs were derived from the unaffected part of liver during resection of benign tumors (hemangioma, cystadenoma) or during extrahepatic pathologies surgery.

## 2. Results

### 2.1. Estimation of Liver Damage Grade and Authentication of MSCs

Biopsy samples from patients with non-malignant neoplasms, cystadenoma, and hemangioma, collected from an undamaged area of the liver, were regarded as clinically healthy liver tissue specimens (Table 1). The study also included samples of pathological liver tissue from patients with clinical diagnoses of fibrosis, cirrhosis, or hepatitis virus (HBV, HCV, HDV) infections (Table 2).

The study included 6 healthy liver samples (5 women and 1 man) the average age of the patients was 45.3 ± 17.5; 2 of the 6 patients were diagnosed with liver cystadenoma, 2 patients with liver hemangioma, and 2 patients with extrahepatic pathologies (pancreatic tail cystadenoma and portal system thrombosis/portal hypertension). The vast majority of hemangioma and cystadenoma cases are not accompanied by system inflammation with exception of rare cases such as giant hemangiomas [21,22].

Biopsies obtained from 12 patients (8 men and 4 women) were used as pathological liver samples. The average age of patients was 41.5 ± 11.2 years (average of 11 patients, data for 1 patient was unavailable); 2 patients out of 12 had liver fibrosis, 6 patients had cirrhosis of viral and/or mixed viral and alimentary-toxic etiology, 3 patients had alcoholic cirrhosis or cirrhosis of unspecified etiology, and 1 patient had an unconfirmed clinical diagnosis of fibrosis or cirrhosis, but was infected with the virus (HBV).

Cells isolated from samples were similar to normal mesenchymal stem/stromal cells by their main phenotypic, morphological, and differentiation properties. MSCs isolated from the liver samples adhered to the plastic; had fibroblast-like morphology (Figure S1); expressed surface MSC markers, including CD29, CD44, CD73, CD90, CD105; and did not express markers of hematopoietic cells, including CD14, CD34, CD45, and also differentiated in the adipogenic or osteogenic lineages in vitro [14].

### 2.2. Phenotypic Characteristics of NL-MSCs and PL-MSCs

**Figure 1:**
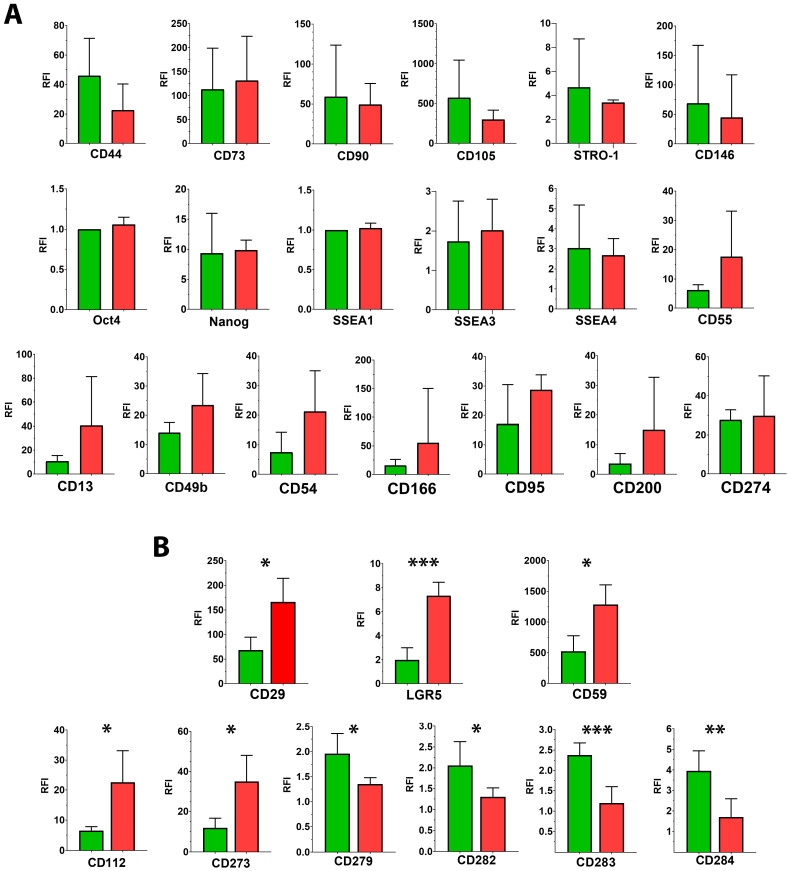
The rates of the expression of markers by NL-MSCs (green) and PL-MSCs (red) represented as the relative fluorescence intensities (RFI). (**A**) Mean (±SD) of RFI for markers without statistically significant differences between NL-MSCs and PL-MSCs groups. (**B**) Mean (±SD) of RFI for markers with statistically significant differences between NL-MSCs and PL-MSCs groups. * *p* ≤ 0.05; ** *p* ≤ 0.01; *** *p* ≤ 0.001.

NL-MSCs and PL-MSCs were compared for the expression of a wide range of surface and intracellular markers characterizing not only the MSC identity, but also pluripotency, adhesive capacity, and immunomodulatory properties. We measured the expression of markers by FACS, followed by the RFI calculation as described in the Materials and Methods (Section 4.2) and applied the two-tailed Student *t*-test for significant differences determination between NL-MSCs and PL-MSCs groups (Figure 1). No less than three different liver MSCs cultures were analyzed in each group for every marker (Figure 1, Table S1). If the RFI was equal to 1, we assumed that there was no expression of the analyzed marker. If the RFI was in the range from 1 to 5, we assumed weak expression of the marker, if in the range from 5 to 10, we assumed moderate expression and if the RFI was greater than 10, we assumed high expression of the analyzed marker.

**Figure 2:**
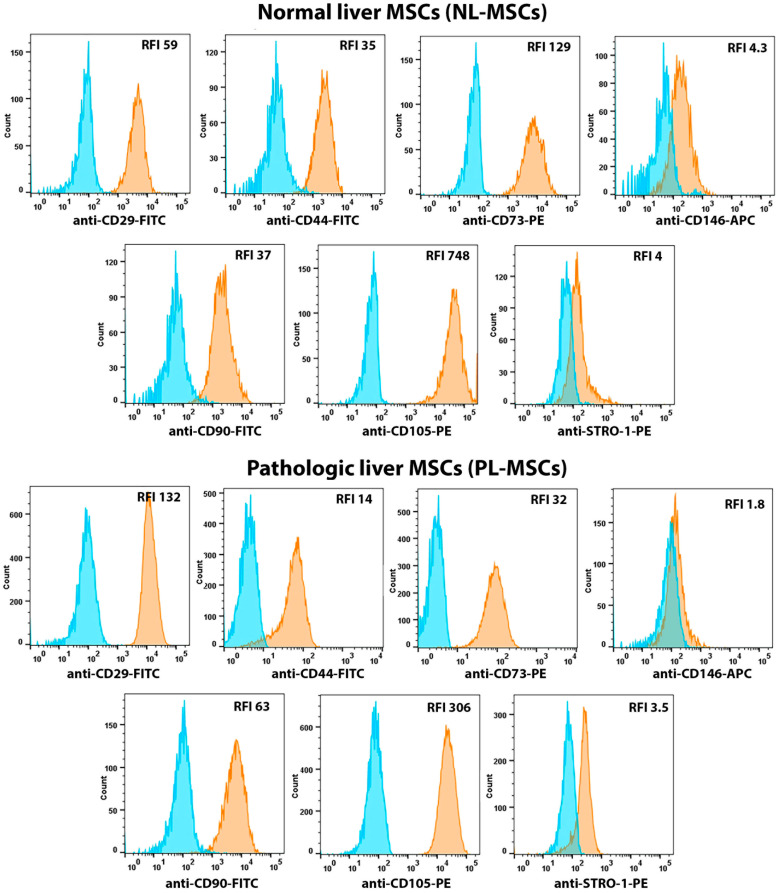
Flow cytometric analysis of mesenchymal cell markers expressed by NL-MSCs and PL-MSCs. Blue peak—autofluorescence of the unstained cells, orange peak—fluorescence of the antibody-stained cells. Representative data.

In this work, the standard spectrum of surface markers common to different types of MSCs including CD29, CD44, CD73, CD90, and CD105 was extended with STRO-1 (stromal precursor antigen-1) and CD146 (Figure 1 and Figure 2, Table S1). Anti-STRO-1 antibodies interact with heat shock cognate 70 (HSC70; HSPA8) on the surface of mesenchymal precursor cells [23]. It was shown that MSCs isolated by immunoselection for STRO-1 had a higher proliferative potential, increased clonogenicity, and the ability to differentiate in vitro compared to plastic adherence-isolated MSCs [24]. CD146 is a marker of pericytes, cells that are considered to be the closest “relatives” to MSCs [25]. However, in addition to pericytes, CD146 has been shown to be expressed by endothelial and smooth muscle cells throughout the vascular tree, regardless of vessel size and anatomical location [26].

No statistically significant differences were found in the expression of mesenchymal markers between the two groups of liver MSCs with the only exception of CD29. The mean RFI for NL-MSCs and PL-MSCs were, respectively: CD29—68.67 and 166.7 (t(4) = 3.13, *p* = 0.035), CD44—46 and 22.67 (t(4) = 1.31, *p* = 0.26), CD73—113.1 and 131.3 (t(4) = 0.25, *p* = 0.81), CD90—59.1 and 49.33 (t(4) = 0.24, *p* = 0.82), CD105—572.7 and 300 (t(4) = 0.97, *p* = 0.39), STRO-1—4.67 and 3.4 (t(5) = 0.65, *p* = 0.55), CD146—68.77 and 45.03 (t(4) = 1.31, *p* = 0.34). Nearly 100% of cells expressed CD29, CD44, CD73, CD90, and CD105 in all analyzed cultures. Expression of STRO-1 and CD146 varied across cultures, and only a proportion of cells in each liver MSC culture expressed these markers. Since CD29 (integrin β1) is part of the integrin receptor complex that interacts with extracellular matrix collagen, its higher expression levels on PL-MSCs may be associated with the altered extracellular matrix in pathological liver, namely with increased collagen levels, compared to normal liver. Thus, MSCs isolated from healthy and pathological liver correspond to the basic characteristics of MSCs obtained from other tissue sources. Namely, they adhere to culture plastic, differentiate into mesodermal cell lineages in vitro [14], and also express a spectrum of surface markers common for MSCs. No statistically significant differences in terms of mesenchymal-related properties were found between NL-MSCs and PL-MSCs.

Maintenance of stemness, as well as the ability of cells to self-renew, is determined by the expression of pluripotency markers, including transcription factors Oct-4, Sox2, Nanog, and surface antigens SSEA-1, SSEA-3, SSEA-4. Stage-specific embryonic antigen-1 (SSEA-1) is a marker of mouse embryonic stem cells (ESCs), but it is not expressed on human ESCs. Human ESCs express SSEA-3 and SSEA-4, while SSEA-1 appears on some differentiated cells [27]. Oct-4 is a POU-domain transcription factor, widely expressed in human ESCs and iPSCs, and its expression is reduced or lost during differentiation. This transcription factor is responsible for maintaining the pluripotent state of cells [28]. Additionally, another transcription factor, Nanog, whose expression has also been shown in ESCs and iPSCs, instead functions as a stabilizer of the pluripotent state, although it is not necessarily required for pluripotency itself [29].

**Figure 3:**
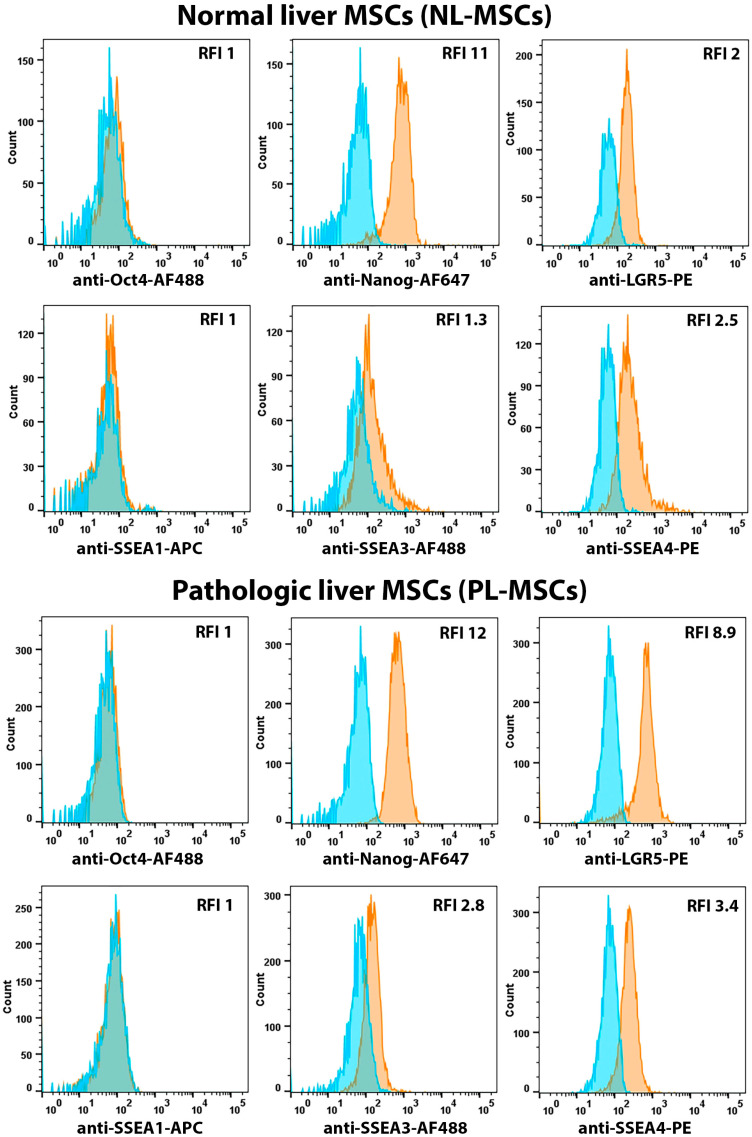
Flow cytometric analysis of the pluripotency markers expressed by NL-MSCs and PL-MSCs. Blue peak—autofluorescence of unstained cells, orange peak—fluorescence of the antibody-stained cells. Representative data.

Expression of transcription factors Oct-4 and Nanog, as well as expression of surface markers SSEA-1, SSEA-3, and SSEA-4, were analyzed in MSCs isolated from healthy and pathological liver in order to assess their “pluripotent” state (Figure 1 and Figure 3, Table S1). None of the analyzed liver MSC cultures expressed Oct-4 and SSEA-1 (RFI for both NL-MSCs and PL-MSCs groups were close to 1), whereas almost 100% of cells in NL-MSC and PL-MSC cultures stained positively with anti-Nanog antibodies. The mean RFI for Nanog expressions in NL-MSCs and PL-MSCs groups were 9.33 and 9.85, respectively, (t(5) = 0.15, *p* = 0.88). Some cells also expressed SSEA-3 and SSEA-4 on their surface (Figure 1 and Figure 3, Table S1). No significant differences were found between MSCs obtained from healthy and pathological livers in the expression of pluripotency markers SSEA-3 and SSEA-4, which may indicate that these cells retain their stem cell properties even under the conditions of developing pathological process. The mean RFI for NL-MSCs and PL-MSCs were, respectively: SSEA-3—1.73 and 2.01 (t(7) = 0.47, *p* = 0.66), SSEA-4—3.03 and 2.68 (t(7) = 0.37, *p* = 0.72). The obtained results may indicate that NL-MSCs and PL-MSCs do not differ in their stemness and pluripotency.

In addition to standard pluripotency-associated markers, we also analyzed the expression of leucine-rich repeat-containing G protein coupled receptor 5 (LGR5) on the surface of liver MSCs. Lgr5 was originally discovered as a stem cell marker in the adult small and large intestine [30], and was later identified as a stem cell marker in adult epithelial tissues, including the stomach [31], mammary glands [32], and ovaries [33]. Currently, Lgr5 expression has been shown on various types of stem cells not only in epithelial tissues, but also on MSCs [34] and resident muscle stem satellite cells [35], and its expression is associated with the regenerative potential of cells.

We showed that the majority of liver MSC populations isolated from both normal and pathological livers express Lgr5 at an intermediate level (RFI 2–8) (Figure 1 and Figure 3, Table S1) with statistical significance between NL-MSCs and PL-MSCs groups (RFI for these groups were 2 and 7.35, respectively, t(5) = 6.57, *p* = 0.001). The function of Lgr5 in MSC is not yet fully understood. It remains unclear whether the expression of this marker on MSCs is a sign of their pluripotency or stem cell state. There is evidence that Lgr5-overexpressing MSCs differentiate in the osteogenic lineage and induce angiogenesis in vitro to a greater extent compared to MSCs with suppressed Lgr5 [34]. In addition, it is known that, in the lungs, Lgr5+ MSCs are localized in alveolar compartments, where they stimulate alveolar differentiation of epithelial precursors, performing the function of the stem cell niche [36]. Thus, it can be speculated that the increased expression of Lgr5 on PL-MSCs compared to NL-MSCs may be associated with their specific stromal function in pathological liver, which is necessary for the maintenance of hepatocytes under chronic inflammation conditions.

MSCs isolated from various tissue sources exhibit increased adhesion to various types of matrices, as well as to other cell types, including vascular endothelium [37]. Cell adhesion is one of the defining mechanisms involved in the migration, proliferation, spreading, and differentiation of MSCs. A wide range of molecules expressed on the cell surface and/or secreted into the microenvironment are involved in MSC adhesion, including integrins, selectins, chemokine receptors, and proteases [38,39].

**Figure 4:**
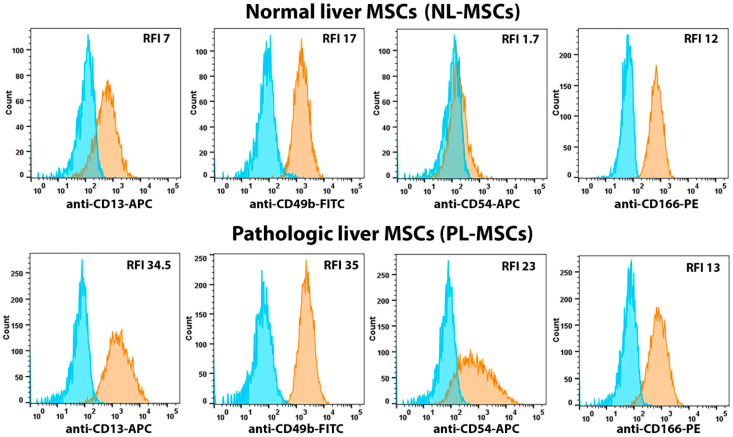
Flow cytometric analysis of the adhesion-associated markers expressed by NL-MSCs and PL-MSCs. Blue peak—autofluorescence of unstained cells, orange peak—fluorescence of antibody-stained cells. Representative data.

On the surface of liver MSCs isolated from healthy and pathological livers, we analyzed the expression of such adhesion molecules as CD49b (Integrin alpha 2), CD54 (ICAM-1), CD166 (ALCAM), and the multifunctional cell surface peptidase CD13, which regulates FAK activation to promote MSC adhesion and migration [40] (Figure 1 and Figure 4, Table S1) and did not find statistically significant differences in the expression of the analyzed adhesion markers in the two groups of MSCs isolated from healthy and pathological liver. The mean RFI for NL-MSCs and PL-MSCs were, respectively: CD13—10.67 and 40.63 (t(5) = 1.23, *p* = 0.27), CD49b—14.13 and 23.5 (t(5) = 1.42, *p* = 0.21), CD54—7.43 and 21.25 (t(5) = 1.57, *p* = 0.18), CD166—15.5 and 55.48 (t(6) = 0.83, *p* = 0.44).

Most cells in both groups expressed relatively high levels of CD13, CD49b, and CD166, whereas CD54 expression varied greatly between different cultures in both groups (RFI from 1.7 to 39). One of the cultures (Liver25) in the PL-MSCs group differed greatly in the expression of the adhesion-associated markers; the RFI values for each marker were several times higher than the RFI values in other NL- and PL-MSCs cultures (Table S1). Such differences are probably associated with the individual characteristics of the biological material from which the cells were obtained.

**Figure 5:**
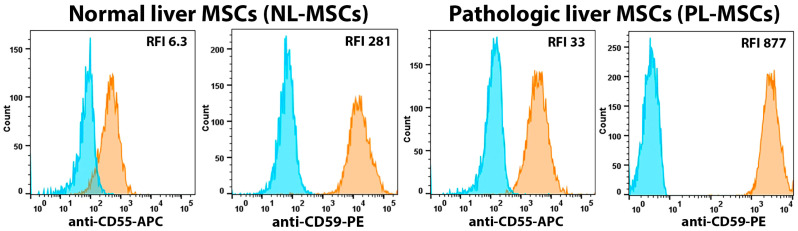
Flow cytometric analysis of the complement-protection markers expressed by NL-MSCs and PL-MSCs. Blue peak—autofluorescence of unstained cells, orange peak—fluorescence of the antibody-stained cells. Representative data.

Many studies have shown that MSCs are resistant to the complement due to the expression of complement-decay accelerating factor (CD55) and protectin (CD59) on their surface [41,42,43]. We showed that liver MSCs isolated from healthy and pathological liver also express high levels of CD55 and CD59 (Figure 1 and Figure 5, Table S1). The expression level of CD59 on liver MSCs was very high (range of RFI was from 281 to 1591) regardless of whether the cells originated from healthy or pathological liver (Figure 1 and Figure 5, Table S1) compared with the expression levels of CD55 on the same cells (RFI from 4.3 to 33). The mean RFI for NL-MSCs and PL-MSCs were, respectively: CD55—6.2 and 17.6 (t(4) = 1.26, *p* = 0.28), CD59—524.7 and 1288 (t(5) = 3.41, *p* = 0.02). Statistically significant difference in expression was shown only for CD59. It can be assumed that the higher expression level of CD59 on PL-MSCs compared to NL-MSCs is an adaptation to the surrounding inflammatory conditions of pathological liver, making them more resistant to complement lysis.

The MSCs potential as therapeutic agents is to a great extent determined by their immunomodulatory properties. MSCs adapt their microenvironment by the expression of the immune checkpoint molecules, thus preventing unwanted immune reactions and maintaining homeostatic immune milieu. In particular, MSCs have been shown to express various isoforms of CTLA-4 (CD152), which are involved in the anti-inflammatory responses [44]. MSCs express PD-1 (CD279), PD-L1 (CD274), and PD-L2 (CD273) and bind to their ligands/receptors on the surface of B cells, T cells, and other cells. PD-1/PD-L1 interaction suppresses T cell proliferation [45]. Recently, such cell surface markers expressed by MSCs as CD39, CD73, CD112, and CD200 have been attributed to checkpoints [46].

**Figure 6:**
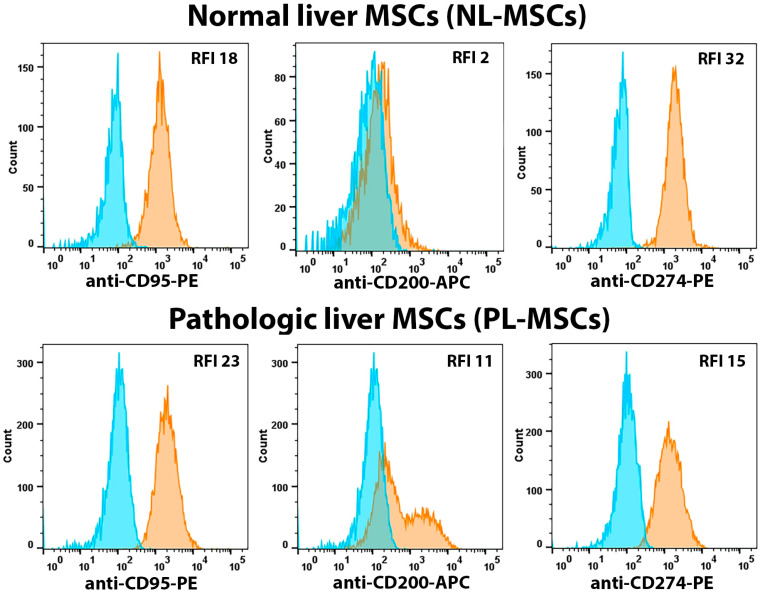
Flow cytometric analysis of immune markers expressed by NL-MSCs and PL-MSCs without different expression. Blue peak—autofluorescence of unstained cells, orange peak—fluorescence of the antibody-stained cells. Representative data.

**Figure 7:**
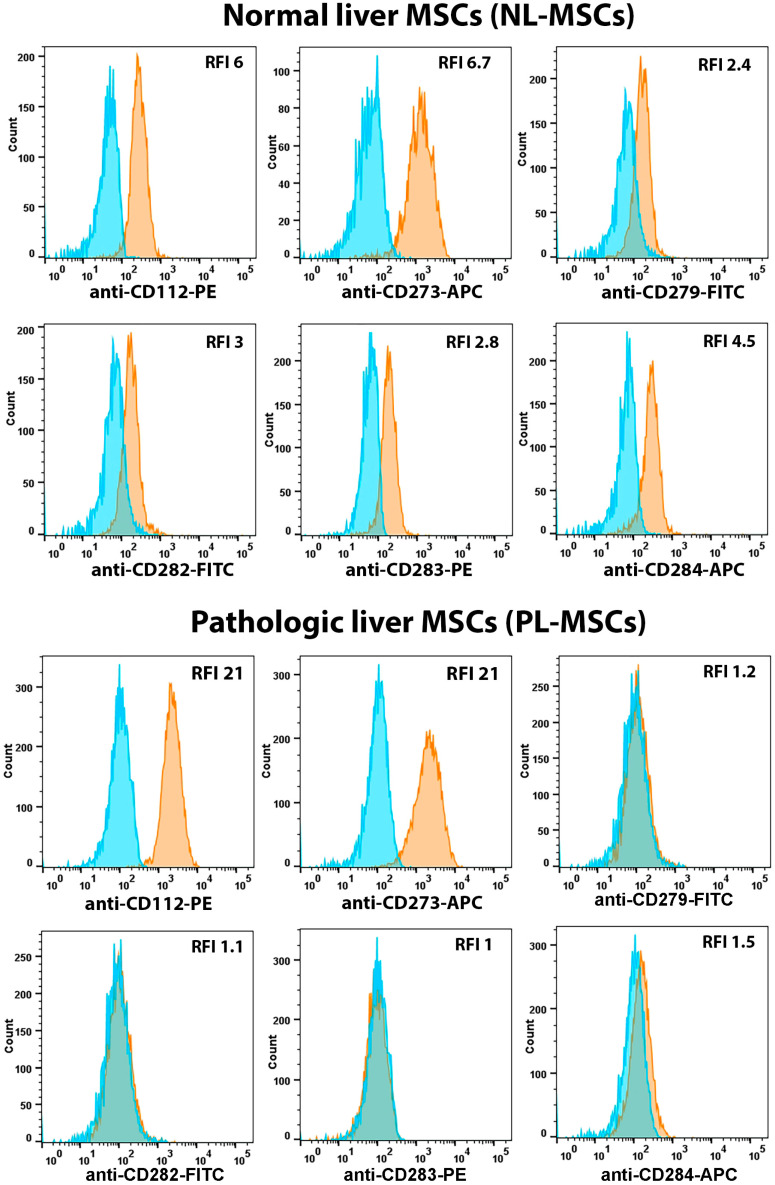
Flow cytometric analysis of immune markers expressed by NL-MSCs and PL-MSCs with different expression. Blue peak—autofluorescence of unstained cells, orange peak—fluorescence of the antibody-stained cells. Representative data.

We analyzed the expression of immune checkpoints including CD112, CD200, PD-1 (CD279), PD-L1 (CD274), and PD-L2 (CD273) on the surface of NL-MSCs and PL-MSCs (Figure 1, Figure 6 and Figure 7, Table S1). We found statistically significant differences in the expression of CD112, CD273, and CD279, but not CD200 and CD274 (Figure 1, Figure 6 and Figure 7, Table S1). The mean RFI for NL-MSCs and PL-MSCs were, respectively: CD112—6.53 and 22.60 (t(5) = 2.56, *p* = 0.05), CD273—11.90 and 35 (t(5) = 2.86, *p* = 0.03), CD279—1.96 and 1.35 (t(7) = 2.87, *p* = 0.02), CD200—3.63 and 15.08 (t(5) = 1.08, *p* = 0.33), CD274—27.67 and 29.75 (t(5) = 0.17, *p* = 0.87). CD200 expression varied widely between cultures, regardless of the cell source. The RFI values for NL-MSCs were around 2, while the RFI values for PL-MSCs ranged from 2 to 41. Almost all cells in the analyzed NL-MSC and PL-MSC cultures expressed CD112, CD273, and CD274. These results suggest that liver-derived MSCs have the potential to utilize immune checkpoint-based immunomodulatory mechanisms.

Also, we found statistically significant differences in the expression of CD282 (TLR2), CD283 (TLR3), and CD284 (TLR4) between normal and pathological liver MSCs. The mean RFI for NL-MSCs and PL-MSCs were, respectively: CD282—2.06 and 1.3 (t(7) = 2.5, *p* = 0.04), CD283—2.38 and 1.2 (t(7) = 5.12, *p* = 0.0014, CD284—3.96 and 1.7 (t(7) = 3.59, *p* = 0.009). Today, it is generally accepted that MSCs isolated from various tissue sources express a wide range of TLRs involved in the regulation of their proliferation, differentiation, motility, and immunomodulatory properties [47]. BM-MSCs, AT-MSCs, and UCB-MSCs were shown to express high levels of TLR3 and TLR4, and low levels of TLR1, TLR2, TLR5, TLR6, and TLR9 [48]. TLR3, TLR2, and TLR4 mediate the immunomodulatory functions of MSCs, including the T-reg activation and immunosuppression [49,50,51].

We found that liver MSCs express relatively low levels of TLR2, TLR3, and TLR4 (Figure 1 and Figure 7, Table S1). Some PL-MSC cultures (Liver2, Liver5, Liver10) did not express TLR3 (CD283; RFI 1), while some (Liver2, Liver5) lacked the TLR4 expression (CD284; RFI 1). However, despite the relatively low overall expression of TLRs by liver MSCs, a statistically significant (*p* ≤ 0.05) difference in TLRs expression was shown between NL-MSCs and PL-MSCs (Figure 1 and Figure 7, Table S1). Thus, it can be speculated that higher levels of PD-1 and TLRs expression on the surface of NL-MSCs may reflect their immunomodulatory activities, probably different from those of PL-MSCs. However, this assumption certainly requires separate research and confirmation in appropriate functional tests.

Along with the immune checkpoints and toll-like receptors, we analyzed the expression of CD95 (Fas receptor). As one of the main molecules that is involved in the apoptosis of transplanted MSCs [14], CD95 also mediates the interaction between immune cells and MSCs [52]. NL-MSCs and PL-MSCs expressed relatively high levels of CD95, and no statistically significant differences were shown between the two cell groups (Figure 1 and Figure 6, Table S1): the RFI means were, respectively, 17.1 and 28.67 (t(4) = 1.4, *p* = 0.24). In addition to the fact that CD95 is a death receptor, the binding of which triggers the apoptotic cascade, the NF-κB signaling pathway was activated in Fas-stimulated MSCs and Fas-induced MSC secretome-mediated immunosuppression [53].

### 2.3. Proliferative and Migration Activities of NL-MSCs and PL-MSCs

Proliferative activity is one of the essential measurable characteristics of MSCs providing the possibility for cell expansion in culture to the quantities required for transplantation and the ability to participate in the reconstituting of various tissues. Its reduction may compromise MSCs regenerative potential, resulting in cell therapy failure [54]. We compared the proliferative activity of NL-MSCs and PL-MSCs by the time-lapse photography of MSC cultures up to the state of cell confluence. Moreover, three cultures of human dermal fibroblast (HDFs) were added for comparison.

**Figure 8:**
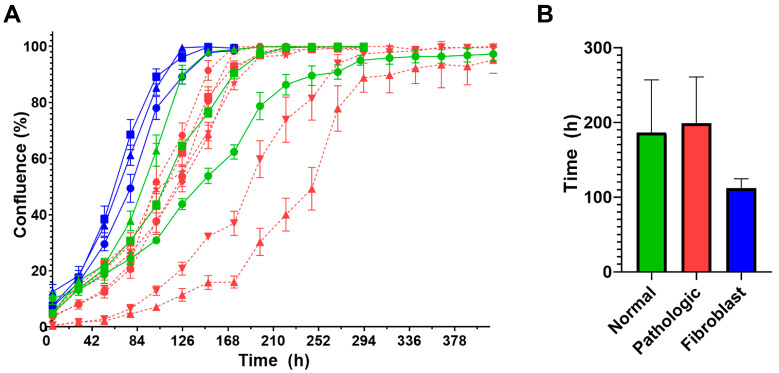
Proliferative activities of NL-MSCs (green, *n* = 3), PL-MSCs (red, *n* = 7), and human dermal fibroblasts (blue, *n* = 3). (**A**) Cell cultures growth curves. Different symbols represent data for the individual cell cultures (mean ± SD). (**B**) Time intervals necessary for reaching confluence by cell cultures (mean ± SD). There are no statistically significant differences between any groups.

A one-way ANOVA test showed no statistically significant difference in the proliferative activities between all the groups (F(2,10) = 2.24, *p* = 0.14). The mean of time intervals required to reach confluence for the NL-MSCs, PL-MSCs, and HDFs cultures were 186, 199, and 112 h, respectively (Figure 8). Importantly, the substantial difference of mean times necessary to achieve confluence between HDFs and the two groups of MSC cultures was statistically insignificant, probably due to limited sample sizes and large spread of values for MSC cultures. Indeed, the variation ranges of time intervals were 126–264 and 144–318 h for NL-MSCs and PL-MSCs cultures, respectively. It highlights the greater importance of the individual features of MSC samples, unrelated directly to liver pathology, such as donor’s genetic features, age, and concomitant diseases, on the proliferative activity of the cells.

Another important MSC feature, the ability to migrate, can be triggered by different stimuli, but primarily by the inflammatory signals. These signals induce MSC migration to the injury site where they modulate the inflammation process intensity and attract other cell populations, contributing to the recovery of tissue integrity. We assessed influence of an inflamed liver environment on the MSCs migration activity and compared the migration abilities of NL-MSCs, PL-MSCs, and HDFs in the scratch-filling experiment using the time-lapse photography.

**Figure 9:**
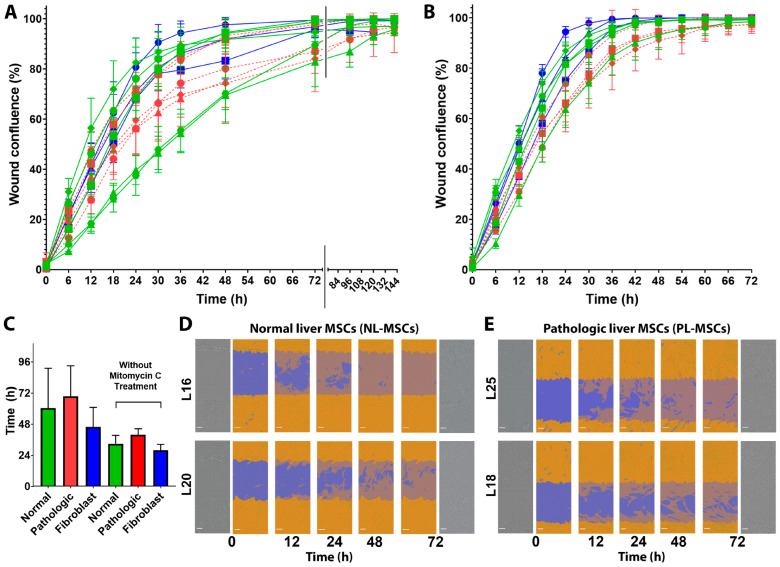
Cell migration ability of MSCs from normal and pathologic liver tissue. (**A**) Wound confluence curves with mitomycin c treatment (NL-MSCs—green, *n* = 5; PL-MSC—red, *n* = 5; HDFs—blue, *n* = 3). (**B**) Wound confluence curves without mitomycin c treatment (NL-MSCs—green, *n* = 4; PL-MSC—red, *n* = 4; HDFs—blue, *n* = 3). Different symbols represent individual cell cultures. (**C**) Mean (±SD) time necessary for reaching confluence by cell cultures in the wound healing assay with and without mitomycin c treatment. There were no any statistically significant differences between NL-MSCs, PL-MSCs, and HDFs groups analyzed separately for mitomycin c treatment cells and without it. (**D**,**E**) Time-lapse brightfield images of wound healing with confluence masks for two NL-MSCs (Liver16 and Liver20) and two PL-MSCs (Liver18 and Liver25) cultures with mitomycin c treatment. The blue mask represents initial wound area, the orange mask represents cell confluence, and merging of both colors represents the initial wound area gradually covered by migrated cells during the healing process. Scale bar—100 µm.

A one-way ANOVA test showed no statistically significant difference in cell migration among all the groups (F(2,10) = 0.82, *p* = 0.47). The mean time necessary for the wound healing was 60.6, 69.6, and 46 h for the NL-MSCs, PL-MSCs, and HDFs cultures, respectively (Figure 9A,C). Without the mitomycin c treatment, those mean time intervals were reduced to 33, 40, and 28 h, respectively (Figure 9B,C), confirming that the scratch closing occurred predominantly by cell migration, not proliferation.

It is important to note that, similar to the proliferative activity, the stretch of the wound closing times in the NL-MSCs and PL-MSCs groups was huge. The time ranges were 39–108 and 45–93 h for NL-MSCs and PL-MSCs groups, respectively. The difference between the slowest (Liver20) and fastest (Liver16) migrating cell cultures from NL-MSCs group was almost three times (Figure 9D). A similar trend was observed in the PL-MSCs group: Figure 9E represents Liver25 and Liver18 cultures with the wound healing times of 45 and 90 h, respectively. This result once again emphasizes the greater impact of the individual donor characteristics compared to the impact of the inflammatory microenvironment onto functional features of the MSCs population.

Despite distinct differences in the proliferative and migratory capacities of individual cultures in both groups, NL-MSCs and PL-MSCs are similar in the basic properties that determine their regenerative and restorative functions.

### 2.4. Senescence Markers in NL-MSCs and PL-MSCs

The aging processes proceed both at the level of the whole organism and at the level of the individual cells. Exhaustion of the stem cell pools and dysfunctional cells accumulated during senescence compromise tissue repair and regeneration. MSCs are no exception [55]. Hereby, we examined the impact of liver inflammation onto the MSC senescence markers [56] by comparing the expression level and activity of senescence-associated beta galactosidase (SA-β-gal), expression of histone γH2AX, and the size of nucleus in NL-MSCs and PL-MSCs.

**Figure 10:**
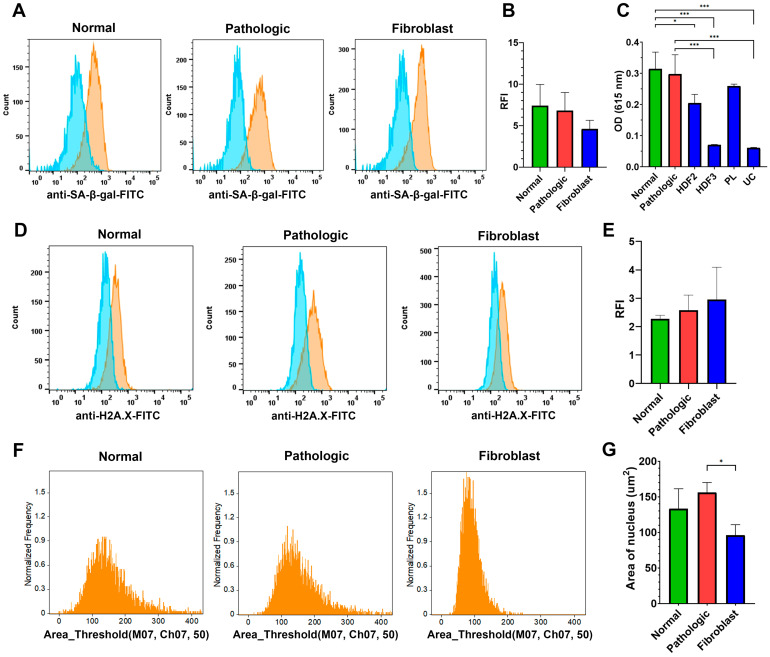
Senescence markers of the MSCs isolated from liver. (**A**) Representative FACS-analysis histograms of senescence-associated beta-galactosidase (SA-β-gal) expression in NL-MSCs, PL-MSCs, and HDFs. (**B**) Mean (±SD) of RFI for SA-β-gal expression in NL-MSCs (*n* = 3), PL-MSCs (*n* = 4), and HDFs (*n* = 3). (**C**) SA-β-gal activity in NL-MSCs (*n* = 5), PL-MSCs (*n* = 3), two human dermal fibroblast cultures—HDF2 and HDF3, and MSCs from placenta (PL) and umbilical cord (UC). For facilitation of graph readability, statistical significance is shown only for the NL-MSCs and PL-MSCs groups. (**D**) Representative FACS-analysis histograms of H2A.X-histone expression in NL-MSCs, PL-MSCs, and HDFs. (**E**) Mean (±SD) of RFI for H2A.X-histone expression in NL-MSCs (*n* = 4), PL-MSCs (*n* = 4), and HDFs (*n* = 3). (**F**) Representative ImageStream distribution of nucleus sizes in NL-MSCs, PL-MSCs, and HDFs. (**G**) Mean values (± SD) of the nucleus area in NL-MSCs (*n* = 5), PL-MSCs (*n* = 3), and HDFs (*n* = 3). * *p* ≤ 0.05; *** *p* ≤ 0.001.

Lysosomal SA-β-gal activity is one of the main markers of cell senescence reflecting the increasing lysosomal biogenesis through aging as a result of accumulation of damaged macromolecules [57]. One-way ANOVA showed no statistically significant differences in SA-β-gal expression between NL-MSCs, PL-MSCs, and HDFs groups (F(2,7) = 1.6, *p* = 0.27). All samples showed equally low expression of SA-β-gal; the specific fluorescence intensity in all samples did not exceed the negative control by more than 10 times (Figure 10A,B).

In line with the SA-β-gal expression, the post hoc Tukey’s multiple comparisons test did not show differences in SA-β-gal activity between NL-MSCs and PL-MSCs groups (*p* = 0.99; Figure 10C), despite the significant difference between six groups by one-way ANOVA (F(5,14) = 28.29, *p* < 0.0001). We showed significant differences in SA-β-gal activity between both NL-MSCs and PL-MSCs on one hand and each of the HDF3 and UC MSCs groups on the other (all *p* < 0.0001), difference between NL-MSCs and HDF2 cultures (*p* = 0.02). Furthermore, we showed significant differences in SA-β-gal activity between the HDF2 and HDF3 groups (*p* = 0.008), HDF2 and UC MSCs (*p* = 0.005), HDF3 and placenta MSCs (*p* = 0.0004), and PL and UC MSCs (*p* = 0.0002).

Cell senescence accelerates inflammation and vice versa [58]. Accumulation of γH2A.X foci occurs during DNA damage and can persist through time, making it a good senescence marker [56,59]. We found no significant differences in γH2A.X foci between the NL-MSC, PL-MSC, and HDF groups by one-way ANOVA (F(2,8) = 0.91, *p* = 0.44; Figure 10D,E).

The size of the nucleus that enlarges during senescence and some pathological processes [60,61] was the last senescence marker assessed in NL-MSCs, PL-MSCs, and HDFs. Similar to other markers, there was no significant difference between the NL-MSCs and PL-MSCs groups in the nucleus area evaluated by the post hoc Tukey’s multiple comparisons test (*p* = 0.38). The only significant difference was between PL-MSCs and HDFs (*p* = 0.02). Initial one-way ANOVA for the three groups was F(2,8) = 5.71, *p* = 0.03.

Thus, we failed to find any statistically significant differences for the checked senescence markers between NL-MSCs and PL-MSCs, suggesting that liver inflammation does not lead to the liver MSC senescence.

### 2.5. NL-MSCs and PL-MSCs Secretome Analysis: Cytokine and Chemokine Multiplex Evaluation

**Figure 11:**
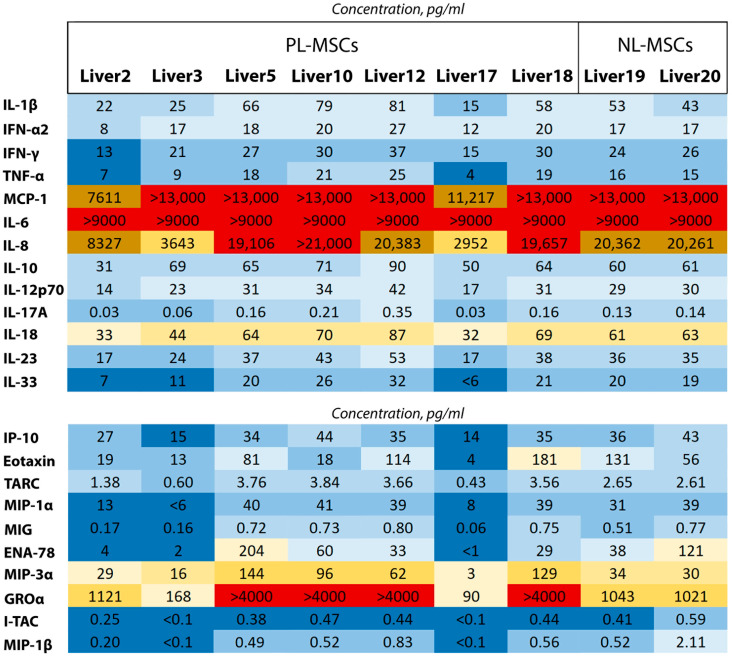
The heatmap representation of the multiplex evaluation of the cytokine (top heatmap) and chemokine (bottom heatmap) secretion by NL-MSCs and PL-MSCs. Different concentrations of molecules are represented by colors: low concentration is represented by blue gradient, high concentration is represented by yellow gradient, highest concentration is represented by red.

Given that the connective tissue expansion resulting in liver damage and failure is clearly linked to inflammation [62,63], our objective was to determine whether MSCs play a role in this process. To characterize the secretion of the proinflammatory mediators in NL-MSCs and PL-MSCs cell culture supernatants, a multiplex assay was used. The multiplex analysis failed to reveal any differences in the secretion of 23 cytokines and chemokines by the cells isolated from pathological and normal tissues (Figure 11). Secretion of the proinflammatory mediators was rather high in both pathological and normal samples, and in some samples the concentration of the analyzed factors exceeded the range of their accurate measurement (in the case of IL-6 for all samples, and in the case of MCP-1 and GROα for some samples). The wide variation in secretion levels between some samples from pathologic livers may be due to the personal characteristics of the patients and the nature of their pathologic process. For example, the concentration of most measured cytokines and chemokines were lower in Liver2 and Liver17 PL-MSC samples than in other measured samples even in the NL-MSC group.

It is noteworthy that the key cytokines constituting the senescence-associated secretory phenotype (SASP), including IL-6, IL-8, and MCP-1 [64,65], were overexpressed in our samples. However, the absence of other senescence markers in both cell types and high proliferative activity of the cells (Figure 8 and Figure 10) indicate that the studied liver MSCs are not in the state of senescence and are unlikely to act as mediators of inflammation. Since the concentrations of the proinflammatory mediators during fibrosis, cirrhosis, and virus infection are known to increase, the serum concentrations of such cytokines as IL-6 and IL-1 are used to measure the severity of liver disease [66,67]. Probably other cells than MSCs contribute to overall high level of the proinflammatory mediators and hepatic inflammation, and even may be responsible for it.

## 3. Discussion

We demonstrated the possibility of obtaining autologous MSCs from the liver of patients with cirrhosis and fibrosis, accompanied by chronic inflammation, and showed that these cells do not differ from healthy liver-derived MSCs in several features, including MSC-related markers, pluripotency markers, proliferation, migration, senescence, spectrum of secreted cytokines, and chemokines.

MSCs definitely play a substantial role in the acute and chronic inflammation, but the underlying cellular and molecular mechanisms have not been fully elucidated. Many studies have shown that inflammatory conditions affect the behavior of MSCs, in particular their secretion profile [68], immunophenotype [69], migratory and proliferative properties, and regenerative potential [70,71]. Importantly, these studies mostly present the results of the in vitro experiments, when MSCs isolated from different tissue sources were exposed to individual proinflammatory factors or their mixture, including IFN-γ, TNF-α, and IL-1β [68]. Most studies using the in vitro inflammation modeling showed that primed MSCs exhibited increased immunosuppressive capacity compared to their untreated counterparts [72,73,74].

Another popular way to create inflammatory conditions in vitro is to stimulate MSCs through the toll-like receptors. TLRs recognize “danger” signals, and their activation leads to cellular and systemic responses that mobilize innate and adaptive immune cells. MSC stimulation via TLR4 induces their switch to a proinflammatory phenotype, which is characterized by high secretion of IL-6 and IL-8 [75]. MSC stimulation via TLR3 results in a switch to an anti-inflammatory phenotype, accompanied by the secretion of IL-4, IDO, and PGE-2 [75,76].

Data concerning the comparison of the properties of MSCs isolated from normal and pathological tissue exposed to inflammatory environment or various danger signals in vivo are less abundant and appear somewhat contradictory to the results obtained in vitro. The main controversy is that the in vitro primed MSCs exhibit increased immunosuppressive potential [72,73,74], whereas most studies comparing MSCs isolated from inflamed tissue and MSCs isolated from normal tissue reported reduced immunosuppressive/immunomodulatory properties of the inflamed tissue-derived MSCs [77,78,79,80]. On the other hand, several studies have shown that despite phenotypic or growth rate differences, MSCs isolated from inflamed or normal tissue exerted similar therapeutic and regenerative effects in animal models of diseases [81,82].

In our work, we did not find differences in the expression of a wide range of markers between MSCs isolated from pathological livers with chronic inflammation (fibrosis, cirrhosis, HBV), and MSCs isolated from healthy livers. The list of stable markers includes the common MSC markers, adhesion molecules, complement protection molecules, markers of the pluripotent state, as well as two immune checkpoints. The obtained results are consistent with the majority of similar studies, which demonstrated the absence of differences in the phenotype of cells isolated from tissues with different degrees of inflammation [77,83]. Interestingly, almost all analyzed cells of NL-MSC and PL-MSC cultures expressed Nanog and did not express Oct-4 (Figure 1 and Figure 3, Table S1). Pierantozzi et al. [84] suggested that the activation of Nanog expression in MSCs in the absence of Oct-4 and SOX-2 expression is associated with the transition from a quiescence state in vivo to adaptation to growth conditions in vitro. The authors found that Nanog was not detected in freshly isolated MSCs, but began to be expressed during cell culture. This factor was detected only in proliferating cells, but not after induction of their differentiation [84]. In amniotic fluid-derived MSCs, Nanog was identified as a key factor in maintaining the cells self-renewal capacity by slowing cellular senescence [85].

We found that PL-MSCs express higher levels of checkpoints such as CD112 and PD-L2 (CD273) compared to NL-MSCs. It has previously been shown that MSCs treated with inflammatory stimuli in vitro express higher levels of CD112 [86] and PD-L2 [87], both of which play an important role in modulating the immunosuppressive effects of MSCs and inducing peripheral tolerance. It is likely that the increased expression of PD-L2 on PL-MSCs may be associated with their presence in pathologically inflamed liver tissue and mediate the immune suppression mechanisms. Yigitbilek et al. [88] showed that hepatic MSCs isolated from brain-dead liver donors significantly reduced NK cell cytolytic function in vitro, mainly due to increased expression and secretion of HLA-C1, which is a ligand for the inhibitory NK receptor KIRDL2/3. On the other hand, it has been shown that MSCs can be lysed by NK cells due to the increased expression of activating NK receptor ligands, including CD112 and CD155 [89]. NK cells, along with other types of innate immune cells, play a key role in regulating inflammation during the development of pathological processes in the liver. NK cell cytotoxicity may contribute to liver injury in various forms of disease. However, NK cells can kill hepatic stellate cell-derived myofibroblasts, thereby limiting liver fibrosis [90]. Increased expression of CD112 on MSCs isolated from pathological liver may be associated with the manifestation of a protective mechanism when it is necessary to activate NK cells for more effective resolution of fibrosis. Thus, it can be hypothetically assumed that in a pathological liver exposed to prolonged inflammation, resident MSCs simultaneously expressing elevated levels of PD-L2 (CD273), which stimulates immunological tolerance (i.e., the adaptive branch of immunity is suppressed), and CD112, which activates NK cells (i.e., the innate branch of immunity), adapt to the microenvironment and perform an immunoregulatory function, and simultaneously continue to play the role of stromal support (chaperone-like) cells.

Phenotypic differences between NL-MSCs and PL-MSCs included higher expression levels of the immune checkpoint PD-1 (CD279) and TLRs including TLR2 (CD282), TLR3 (CD283), and TLR4 (CD284) on the NL-MSC surface. In the liver, TLRs play an important role in maintaining adequate responses to pathogens and danger signals. They recognize not only molecular patterns of pathogens, but also interact with damage-associated molecular pattern molecules (DAMPs) produced as a result of liver cell damage. Given the constant exposure of the liver tissue to pathogens from the gut, strict control of the TLR-related signaling pathways is required in the liver in order to prevent inappropriate production of proinflammatory cytokines and triggering the development of autoimmune and chronic inflammatory diseases [91]. Due to certain specific functions of liver cells and the distribution of TLRs, liver is more a site of tolerance rather than immunity. In the liver, TLR2, TLR3, and TLR4 are expressed by hepatocytes, Kupffer cells, and biliary epithelial cells. Liver sinusoidal endothelial cells express TLR3 and TLR4; hepatic stellate cells express TLR4 [92]. In the alcoholic liver disease, there is evidence that TLR2 deficiency significantly alleviates damage, while TLR3 deficiency, on the contrary, aggravates it [93]. Deficiency or mutation of TLR4 in the alcoholic liver disease and in the liver fibrosis models alleviates the symptoms of the disease due to reduced production of proinflammatory mediators and no free radical production, which results in fewer fatally damaged hepatocytes [94]. In normal conditions and during the development of a pathological process in the liver, the expression and activation of TLRs clearly are orchestrated to maintain the inherent regenerative potential of the organ and to preserve the immune homeostasis. It is likely that the reduced expression of TLRs on MSCs isolated from pathological liver may be associated with a certain regulatory mechanism, which, by reducing the expression of receptors, also reduces the net signal triggered by their activation, preventing excessive activation of cells in the proinflammatory format. Of course, this is a purely speculative assumption that requires experimental confirmation. Since the role of the resident MSCs in the liver remains unclear, it is difficult to accurately evaluate the extent of their participation in the development of pathological processes and/or the implementation of normal physiological functions of the liver.

A specific regulatory mechanism controlling the expression of PD-1 on resident liver MSCs under the inflammatory conditions is also a possibility. The PD-1 inhibitory pathway plays a significant role in the development of pathological processes in the liver. PD-1/PD-L1 signaling is involved in the regulation of the responses of T cells in acute and chronic liver inflammation, and also participates in the expansion of inflammation in liver diseases [95]. The role of PD-1 expression by MSCs is not yet clear. In a single study [96], it was shown that the neural crest-derived MSCs from the dental pulp (MSC-DP), but not MSCs from the bone marrow, expressed PD-1, and this receptor acts as a key surface molecule controlling the proliferation and differentiation of MSC-DP in vitro. Taking into account that the abovementioned studies demonstrated a reduced immunomodulatory capacity of MSCs isolated from inflamed tissues, we can also assume that the difference in PD-1 and TLRs expression between PL-MSCs and NL-MSCs potentially indicates differences in the immunomodulatory properties of these cells. However, this assumption also requires additional studies.

Reducing of the proliferative activity is probably one of the main limitations for MSCs involvement in the regeneration processes [55]. We compared proliferation of NL-MSCs and PL-MSCs and did not find any significant differences (Figure 8). The spread in time intervals necessary for reaching monolayer confluence in both groups was very high. Apparently, the proliferation activity of analyzed MSCs was not associated with the age of the donor: for example, PL-MSC sample Liver12 with longest time for reaching confluence was derived from a 29 years old patient. Other individual features of donors beyond the age might contribute to the vast spectrum of MSCs proliferative activities.

Moreover, we obtained similar results with the NL-MSCs and PL-MSCs motility: there was no statistically significant differences in their migration properties (Figure 8). It is important to note that realization of healing properties of MSCs involves its migration to the inflammation foci [97]. Different types of MSCs possess different migration ability: for example, Hori et al. showed higher migration ability of the UC-MSCs compared to the BM-MSCs and AT-MSCs [98]. Furthermore, MSCs migration properties and their homing to injured tissues are orchestrated by various soluble factors and depend on the severity of inflammation [99]. As for proliferation activity, we demonstrated high dispersion of the migration ability in both groups, NL-MSCs and PL-MSCs, again lacking clear association with the age of donor: the slowest PL-MSCs cultures Liver2 and Liver18 (wound healing times 93 and 90 h, respectively) were both derived from the 37-year-old donors (Table 1 and Figure 9).

Thus, we showed that the two major functional properties of MSCs—proliferation and migration—stay apparently unaltered during chronic liver inflammation. Perhaps even during severe inflammation such as cirrhosis or viral hepatitis, liver MSCs continue to contribute to the maintenance of a stable microenvironment. Furthermore, dispersion in MSCs properties in normal and pathologic groups is apparently dependent on individual characteristics of donors, including concomitant diseases and genetic features, but not age. Of course, these conclusions need to be verified by studies performed with larger sample sizes and using additional methods.

Chronic inflammation and cellular senescence are interconnected. In many tissues, the aging processes switch the microenvironment to the proinflammatory mode [58] while some chronic diseases exhaust different cell population including adult stem cells [100]. We decided to estimate and compare the «senescence level» of MSCs isolated from the liver undergoing chronic inflammation and MSCs from healthy liver tissue. We chose three markers of cellular senescence most used for discrimination of skin fibroblasts taken from young and old donors: SA-β-gal activity and expression, the level of γH2A.X, and the size of cell nucleus [56]. We showed no statistical differences between these parameters in the NL-MSCs and PL-MSCs groups. Hence, chronic inflammation is unlikely to affect MSCs pool integrity and does not lead to the senescence-associated phenotype, again stressing the stability of liver MSCs under the inflammatory conditions.

Despite the fact that it is believed that MSCs primarily exhibit immunomodulatory properties, there are works showing their ability to provoke inflammation by secretion of the various mediators [101,102,103]. The multiplex analysis demonstrated a high degree of similarity of the secretion profiles of MSCs isolated from normal and pathological tissues, which casts doubt on the involvement of liver MSCs in promoting the organ inflammation. It seems plausible that other cell populations may play a role in the pathological process. For example, hepatic stellate cells, capable of producing proinflammatory soluble mediators and differentiate into myofibroblasts are clearly candidates for this role [104]. As illustrated in Figure 11, not only PL-MSCs but NL-MSCs exhibit a considerable degree of proinflammatory mediator secretion, which leads to the conclusion that this is the natural level of these mediator secretion for the liver MSCs. Apparently, this is not unique to this tissue. For example, MSCs isolated from normal adipose tissue secreted amounts of MCP-1 and IL-6 similar to those obtained in our experiments with liver MSCs, and the secretion of IFN-γ and TNF-α in the same samples was even markedly higher [105]. This issue becomes very important in the context of the potential therapeutic use of the MSCs secretome [106]. Nevertheless, it is very likely that many MSCs types are characterized by a proinflammatory secretion profile even under normal tissue conditions. Concurrently, it is evident that exposure of MSCs to such cytokines as IL-1β, IFN-γ, and TNF-α can activate these cells and markedly enhance their secretion of proinflammatory cytokines [107]. Importantly, the proinflammatory profile of MSC secretion does not necessarily imply their involvement in stimulation of the immune reactivity. To the contrary, such MSCs instead have immunosuppressive properties. In our study of the endometrial MSCs, only cells with a pronounced proinflammatory secretion profile exhibited robust immunosuppressive capabilities, as evidenced by their capacity to inhibit dendritic cell differentiation and lymphocyte proliferation in vitro [103]. Furthermore, MSCs derived from human umbilical cord, despite their proinflammatory secretion profile, completely suppressed dendritic cell differentiation [108] and the cytotoxic activity of NK cells [109]. This aligns with the notion that certain cytokines, including IL-6, may possess both pro- and anti-inflammatory properties in different cell types and microenvironments [110].

The results obtained in this work largely agree with some published data regarding the similarity of the phenotype, secretome, and functions of MSCs isolated from inflamed and normal tissues. It is worth noting that even in the chronically inflamed liver, a pool of functionally active MSCs is preserved, which potentially have proregenerative properties common to MSCs from normal tissues. It is not yet clear in what quantity MSCs are present in the liver during long-term chronic inflammation and the development of end-stage liver disease and whether they can be expanded in vitro to the quantities required for cell therapy.

The main limitation of the obtained results is the insufficient sample size of liver samples in each group. Although statistically significant differences in proliferation, migration, senescence, and chemokine/cytokine secretion were not found between MSCs from pathological and healthy liver, it is worth noting that peculiar differences in individual MSC cultures in each group were observed. To reliably establish whether these differences are associated with individual patient characteristics or with the stage of the disease or other external and/or internal factors, a larger sample of patients with more stringent inclusion criteria is required. The differences in the expression of immune checkpoints and TLRs found between NL-MSCs and PL-MSCs require further confirmation in functional tests and in vivo experiments. For now, we can only speculate that the different expression of these markers may indicate differences in some properties of MSCs.

## 4. Materials and Methods

### 4.1. Isolation and Cultivation of Human Liver MSCs and Other Primary Cultures

Liver biopsies were provided by the Petrovsky Russian Research Center of Surgery within the framework of the project Unique Scientific Installation “Avogadro”. The study was approved and supervised by the Institutional Ethics Committee (Protocol No. 4, 21 April 2022). Written informed consent was obtained from all patients. Biological samples were handled using anonymous codes in accordance with the Federal Law on Personal Data (No. 152-FZ, 27 July 2006). Pathological liver biopsies were obtained during the azygo-portal disconnection operations [111]. Healthy liver samples were obtained by collecting unaffected liver tissue during the operations performed to remove non-cancerous neoplasms [22,112].

Immediately after the operation, the biopsy sample was placed in a tube containing Hanks’ solution supplemented with an antibiotic/antimycotic and stored at +4 °C. All liver samples were delivered to the laboratory within 24 h after surgery, maintained at +4 °C during the transfer. Further processing of liver tissue samples was carried out according to a proven standardized method [84]. In brief, liver tissue samples were minced and incubated in type IV collagenase solution (0.1% *w*/*v*; Gibco, Waltham, MA, USA) for 30 min. Cell suspension and remaining liver tissue were washed in PBS (Gibco, Waltham, MA, USA) supplemented with 1% FBS (Gibco, Waltham, MA, USA) and centrifuged, and the pellet was resuspended in the complete growth medium composed of DMEM/F12, 10% FBS, 100 U/mL penicillin, and 100 μg/mL streptomycin (all reagents from Gibco, Waltham, MA, USA). Adherent cells had fibroblast-like morphology, formed a monolayer and were photographed using a Nikon D5000 camera (Nikon, Tokyo, Japan; Figure S1). Cell morphology was assessed using a Zeiss Axiovert 40CFL phase-contrast microscope (Carl Zeiss, Jena, Germany).

All other cell cultures, including human dermal fibroblasts (HDF) and different MSC cultures were provided by the laboratory of cell biology (Institute of Biomedical Chemistry, Moscow, Russia). Cell cultures were maintained in the complete growth medium in a CO_2_ incubator (37 °C, 5% CO_2_, 80% humidity). Upon reaching 80–90% confluency, the cells were passaged. In all experiments, the cells from no later than the 10th passage were used.

### 4.2. Flow Cytometry

The cells were trypsinized and washed twice in PBS (PanEco, Moscow, Russia) supplemented with 1% FBS (Gibco, Waltham, MA, USA) by centrifugation for 5 min at 300× *g*. The cells were incubated with appropriate antibodies to surface antigens (Table 3; BD Biosciences, Franklin Lakes, NJ, USA) at concentrations recommended by the manufacturer for 1 h at 4 °C in the dark.

To stain intracellular antigens, the cells after trypsinization were fixed with BD Cytofix fixation buffer (BD Biosciences, Franklin Lakes, NJ, USA), permeabilized with 0.1% Triton (10 min), incubated with antibodies to corresponding intracellular markers (Table 3) for 1 h, and, if applicable, with secondary anti-species antibodies for another 1 h. Then, the cells were washed and fixed with BD Cytofix fixation buffer (BD Biosciences, Franklin Lakes, NJ, USA). Fluorescence intensity was analyzed using the BD FACSAria III flow cytometer (BD Biosciences, Franklin Lakes, NJ, USA). At least 10^4^ events were recorded in each sample. The results were processed using the FlowJo_V10 (FlowJo™, Ashland, OR, USA). The relative fluorescence intensity (RFI) representing the marker expression was calculated as the ratio of the specific fluorescence of cell staining with fluorescently labeled antibodies and the autofluorescence of control unstained cells. In the case of the indirect cell staining with secondary antibodies, cells stained with secondary antibodies only were used as the control.

### 4.3. Imaging Flow Cytometry

Imaging flow cytometry was performed using an Amnis ImageStreamX Mk II Imaging Flow Cytometer (Luminex Corporation, Austin, TX, USA) and all data were analyzed using IDEAS software (Version 6.2). Cell suspensions were fixed with BD Cytofix fixation buffer (BD Biosciences, Becton Drive Franklin Lakes, NJ, USA) and stained with Hoechst 33,342 (Thermo Fisher Scientific, Waltham, MA USA) at the final concentration of 10 µg/mL. Primary gating strategy included gating of focused cells using the «Gradient RMS» feature in the brightfield channel (top 50% of cells by this feature), followed by gating single cells using the «Area»-«Aspect Ratio» in the same channel. The cell nucleus areas were calculated using the Threshold mask with 50% intensity percentage in the channel 07 (Area_Threshold (M07, Ch07, 50)).

### 4.4. Cell Proliferation Assay

Liver MSCs and HDFs were plated in the 12-well plates at the 10,000 cells per well density. The Incucyte ZOOM system (Sartorius, Gottingen, Germany) was used to capture the cell growth phase images and their further analysis (software version—2016A). Sixteen or twenty-five phase images per well were obtained every 6 h using the 10× or 20× objective, respectively. «Job analysis» protocol was applied to the images using the following parameters: segmentation adjustment—1.3, hole fill—700 µm^2^, filters—minimum 500. The monolayer was considered complete after achieving 90% confluence.

### 4.5. Cell Migration Assay

Liver MSCs and HDF cells were seeded into the Incucyte Imagelock 96-well plate (Sartorius) using full culture media with a 5 × 10^5^ cells per well density. Cell monolayer was treated for 2 h with 10 μg/mL mitomycin C (Merck, Rahway, NJ, USA) to suppress cell proliferation and scratches were inflicted with the IncuCyte 96-pin wound-making tool (Sartorius). The Incucyte ZOOM system (Sartorius), was used to capture the cell migration phase images every 1.5 h using the 20× objective and further analysis of confluence (software version—2016A). Job analysis was applied for images using the following parameters: segmentation adjustment—1.3, hole fill—700 µm^2^, filters—minimum 500. Wound confluence was calculated for each image. The monolayer was considered complete after achieving 90% confluence.

### 4.6. Senescence-Associated β-Galactosidase (SA-βGAL) Assay

To assess β-galactosidase activity in liver MSCs, skin fibroblasts, and MSCs isolated from placenta and umbilical cord, cells were seeded into wells of a 96-well plate (40 thousand cells/well) in complete growth medium, each culture in triplicate. After complete adhesion, cells were fixed in 0.2% glutareldahyde for 15 min at room temperature. Cells were washed twice in PBS and X-gal solution (1 mg/mL) was added and incubated overnight at 37 °C. The next day, the staining solution was aspirated, cells were washed twice in PBS, and DMSO was added to the wells. The supernatant was transferred to a new plate. Optical density measurements were performed on a Tecan infinite M200 Pro plate reader (Tecan, Männedorf, Switzerland) at the 615 nm wavelength.

### 4.7. Cytokine and Chemokine Evaluation

The following two bead-based multiplex assays were used for cytokine and chemokine evaluation in the liver MSCs culture supernatants: Legendplex, Human Inflammation Panel 1 (Cat# 740808; BioLegend, San Diego, CA, USA), and Chemokine Panel 1 (Cat#740984, BioLegend). All staining and analyzing procedures were performed according to the manufacturer’s recommendations. Briefly, assay buffer, beads, and standard or analyzed samples were mixed and incubated for 2 h in a filter plate, washed using vacuum filtration unit, and stained with detection antibodies for 1 h. After stained beads incubation with streptavidin–phycoerythrin for 30 min, all samples were initially analyzed using the FACSAria III instrument with BD FACSDiva software (Version 8.0, BD Biosciences) in duplicate. The LEGENDplex Data Analysis (2024) Software Suite was used for analyte concentration evaluation and heatmap composition.

### 4.8. Statistical Analysis

GraphPad Prism 8.0 software was used for statistical analysis and graph creation. The applied statistical methods are described in the Section 2. The value of *p* ≤ 0.05 was considered statistically significant.

## 5. Conclusions

In this work, we have shown that MSCs isolated from pathological liver characterized by chronic inflammation are very similar to MSCs isolated from healthy liver, based on their fundamental cellular properties, including proliferation, migration, and senescence, as well as secretion of the proinflammatory cytokines and chemokines (IL-6, IL-8, MCP-1, and GROα). These results may indicate that resident MSCs remain in their functional state and are capable of performing supporting chaperone-like functions regardless of the inflammatory status of the liver, probably within regenerative nodules formed in the liver during cirrhosis. The pool of resident MSCs is maintained until the end-stage liver disease. Despite their proinflammatory status, the liver MSC secreted cytokines, such as IL-6 and IL-8, also perform a regenerative function during acute and chronic liver inflammation. IL-6 is a potent mitogen for hepatocytes [113], and IL-8 is able to regulate the conversion of mature hepatocytes into cholangiocytes, thereby replenishing the pool of bipotent liver progenitor cells [114].

The phenotypic differences we found between NL-MSCs and PL-MSCs mostly concern immune checkpoints and TLRs. It is possible that these differences may mediate the different immunomodulatory properties of MSCs isolated from the healthy and pathological liver. However, it seems equally likely that the mechanisms of immunomodulation under chronic inflammatory conditions differ from those under normal non-inflammatory conditions. Both of these assumptions require further confirmation in appropriate experiments.

Our results suggest that even in the presence of chronic liver inflammation, isolating and expanding autologous liver MSCs for subsequent autotransplantation to a patient with liver disease is a realistic task. Quite likely, the transplantation can invoke a positive therapeutic effect, since, firstly, liver MSCs are liver-committed, and secondly, they are already adapted to the current inflammatory conditions. Of course, there may be various clinical limitations for such autotransplantation that still need to be established. Confirmation of the therapeutic efficacy and safety of these cells in functional tests is also required. On the other hand, the detection of MSCs in pathological liver samples that are almost similar in their properties to MSCs from normal liver opens new opportunities for developing methods for stimulating and/or activating their proregenerative properties in situ. From the point of view of the potential of tissue regeneration, the presence of virtually normal MSCs in pathological liver appears encouraging.

## Figures and Tables

**Table 1:** Clinical data concerning healthy liver samples.

№	Sample	Sex/Age	Diagnosis
1	Liver15	W/46	Liver cystadenoma, hypertension stage 1, obesity class 3
2	Liver16	W/46	Liver hemangioma, scleroderma, varicose veins of both lower extremities
3	Liver19	W/41	Liver cystadenoma
4	Liver20	W/76	Pancreatic tail cystadenoma, atherosclerosis, hypertension stage 2, chronic gastritis
5	Liver22	W/22	Thrombosis and cavernous transformation of the portal vein, secondary extrahepatic portal hypertension, gastroesophageal varices grade 3, threat of gastrointestinal bleeding
6	Liver24	M/41	Liver hemangioma

**Table 2:** Clinical data concerning pathologic liver samples.

№	Sample	Sex/Age	Diagnosis
1	Liver2	W/37	Liver fibrosis (F1-2). Thrombophilia (hyperhomocysteinemia, heterozygous polymorphism of the methionine synthetase reductase genes, MTHFR, PAL-I). Thrombosis and cavernous transformation of the trunk and lobar branches of the portal vein, splenic and superior mesenteric veins. Extrahepatic portal hypertension. Esophageal varices grade 3. Ascites grade 1.
2	Liver3	M/48	Liver cirrhosis mixed (viral HCV and alimentary-toxic) etiology. Compensated: Child–Puqh class A. Portal hypertension. Gastro-esophageal varices GOV 2 grade 3. Erosive antrum gastritis.
3	Liver5	M/53	Liver cirrhosis mixed (viral HCV and alimentary-toxic) etiology. Compensated: Child–Puqh class A. Portal hypertension. Gastro-esophageal varices GOV 2, grade 3. Axial hiatal hernia grade 3. History of recurrent bleeding.
4	Liver10	W/23	Liver fibrosis (F1-2), portal vascular anomaly, primary extrahepatic portal hypertension. Gastro-esophageal varices, grade 3. Recurrent bleeding. Congenital thrombophilia (Leiden factor).
5	Liver11	W/47	Liver cirrhosis, viral HCV and HDV etiology. Child–Puqh class B. Portal hypertension. Esophageal varices grade 1 and stomach varices GOV 2 grade 3. Ascites. Splenomegaly. “Hypersplenism”. Gastro-esophageal bleeding. Posthemorrhagic anemia.
6	Liver12	M/29	Liver cirrhosis, viral HCV etiology. Child–Puqh class AB. Portal hypertension. Esophageal varices grade 2–3 and gastric varices GOV 2 grade 2–3. Recurrent gastro-esophageal bleeding.
7	Liver13	W/28	Liver cirrhosis, alimentary etiology. Child–Puqh class A. Portal hypertension, esophageal varices grade 2–3 and gastric varices GOV 2 grade 3. Recurrent gastrointestinal bleeding.
8	Liver17	M/53	Liver cirrhosis, viral HCV etiology. Child–Pugh class A. Portal hypertension, esophageal varices grade 3 and stomach varices GOV 1 grade 3. Established gastrointestinal bleeding.
9	Liver18	M/37	HBV virus. Thrombosis of the portal vein and its branches. Extrahepatic portal hypertension. Esophageal varices grade 3 and stomach varices grade 3. Recurrent gastrointestinal bleeding.
10	Liver21	M/*	Liver cirrhosis mixed (HCV/HBV and alimentary-toxic) etiology.
11	Liver23	M/54	Unspecified liver cirrhosis. Thrombophilia, total thrombosis of the portal system, esophageal varices grade 1, stomach IGV 2 grade 3 and duodenal bulb grade 2. Recurrent gastrointestinal bleeding.
12	Liver25	M/48	Liver cirrhosis, alimentary-toxic etiology. Child–Pugh class B (7 points), Meddrey index 17.9%. Portal hypertension. Esophageal varices grade 3, stomach varices GOV 1 grade 2. Recurrent gastrointestinal bleeding. Mixed anemia. Ascites Grade 1 (IAC). Small-nodular liver cirrhosis with signs of pronounced stromal activity.

* Data unavailable.

**Table 3:** List of antibodies for FACS analysis used in work.

№	Name	Company	Cat. №
1	APC Mouse Anti-Human CD29	BD Pharmingen (Becton Drive, Franklin Lakes, NJ, USA)	559883
2	FITC Mouse Anti-Human CD44	BD Pharmingen	555478
3	PE Mouse Anti-Human CD73	BD Pharmingen	550257
4	FITC Mouse Anti-Human CD90	BD Pharmingen	555595
5	PE Mouse anti-Human CD105	BD Pharmingen	560839
6	APC Mouse Anti-Human CD13	BD Pharmingen	557454
7	FITC Mouse Anti-Human CD49b	BD Pharmingen	555498
8	APC Mouse Anti-Human CD54	BD Pharmingen	559771
9	PE Mouse Anti-Human CD166	BD Pharmingen	559263
10	APC Mouse Anti-Human CD55	BD Pharmingen	555696
11	APC Mouse Anti-Human CD95	BD Pharmingen	558814
12	APC Anti-Human CD146	BioLegend (San Diego, CA, USA)	361016
13	PE Anti-Human CD112 (Nectin-2)	BioLegend	337409
14	APC Anti-Human CD200 (OX2)	BioLegend	399808
15	APC Anti-Human CD273 (B7-DC, PD-L2)	BioLegend	345508
16	PE Anti-Human CD274 (B7-H1, PD-L1)	BioLegend	329706
17	FITC Anti-Human CD279 (PD-1)	BioLegend	329904
18	FITC Anti-Human CD282 (TLR2)	BioLegend	309705
19	PE Anti-Human CD283 (TLR3)	BioLegend	315009
20	APC Anti-Human CD284 (TLR4)	BioLegend	312815
21	Alexa Fluor^®^ 488 Anti-Oct4 (Oct3)	BioLegend	653706
22	Alexa Fluor^®^ 647 Anti-Nanog	BioLegend	674210
23	PE Anti-Human LGR5 (GPR49)	BioLegend	373804
24	APC Anti-Human CD15 (SSEA-1)	BioLegend	301908
25	Alexa Fluor^®^ 488 Anti-Human/Mouse SSEA-3	BioLegend	330306
26	PE Anti-Human SSEA-4	BioLegend	330406
27	PE Anti-Human CD59	BioLegend	304707
28	PE Anti-STRO1	Abcam (Cambridge, UK)	ab190282
29	FITC-Linked Polyclonal Antibody to Galactosidase Beta (GLb)	Cloud-Clone Corp. (Katy, TX, USA)	LAA196Hu81
30	Rabbit Anti-Phospho-Histone H2A.X (Ser139)	Cloud-Clone Corp.	AF3187
31	Goat Anti-Rabbit IgG (FITC)	Abcam	ab6717

## Data Availability

The data presented in this study are available within the article text, figures, tables, and Supplementary Materials.

## Supplementary Materials

The following supporting information can be downloaded at: https://www.mdpi.com/article/10.3390/ijms252413374/s1.
