# Supplementary material for: Human Liver MSCs Retain Their Basic Cellular Properties in Chronically Inflamed Liver Tissue
Source: Int J Mol Sci. 2024 Dec 13;25(24):13374. doi: 10.3390/ijms252413374 (PMC11676302; doi:10.3390/ijms252413374)

Figure S1. MSCs morphology assessment on initial steps of isolation. Bar scales: 25 µm.

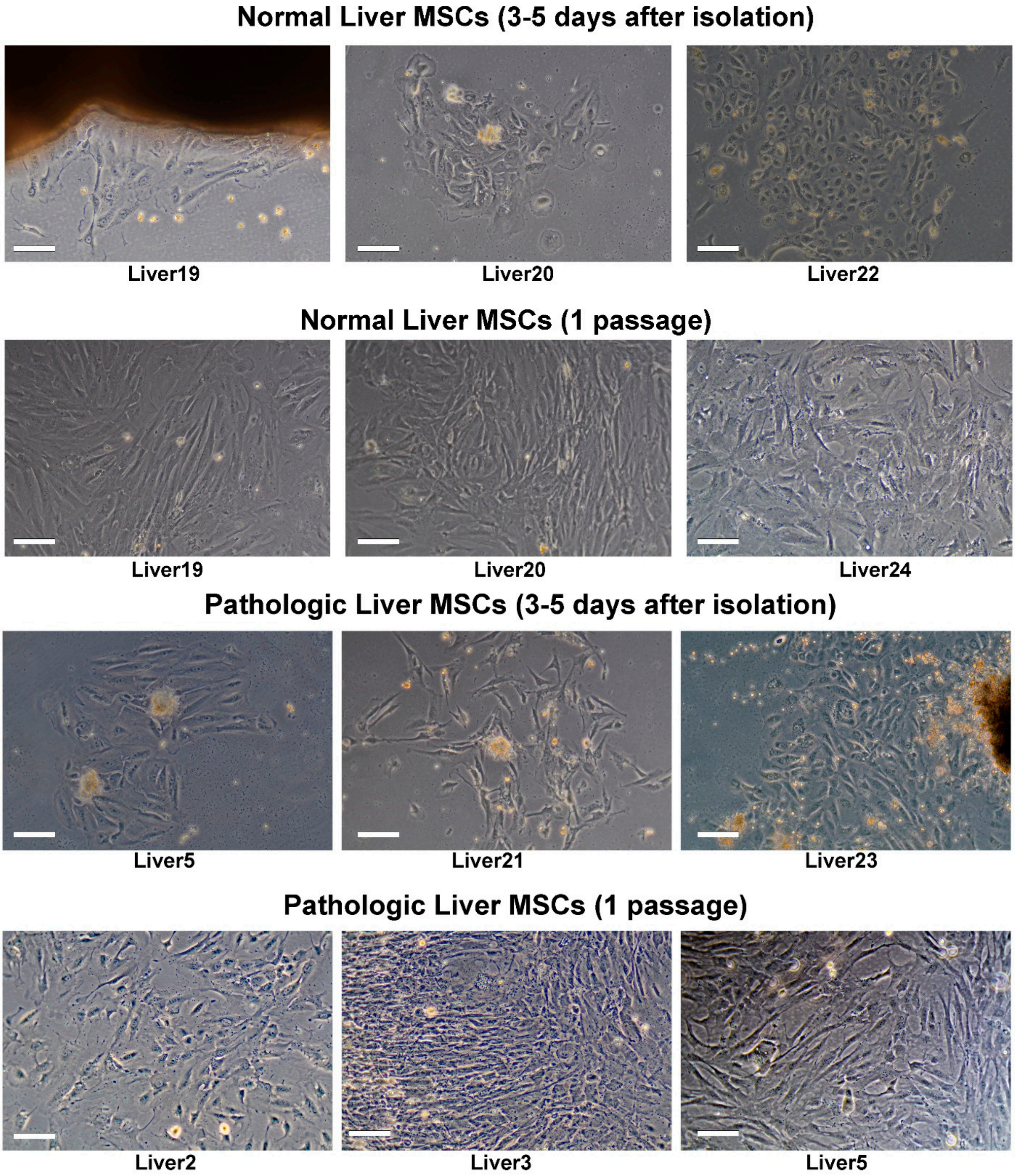

Supplement: Supplementary file 1 [file ijms-25-13374-s001.zip › Supplementary Figure S1.pdf]
